# Dry Friction and Wear Behavior of Laser-Sintered Graphite/Carbon Fiber/Polyamide 12 Composite

**DOI:** 10.3390/polym15193916

**Published:** 2023-09-28

**Authors:** Abdelrasoul Gadelmoula, Saleh Ahmed Aldahash

**Affiliations:** 1 Department of Mechanical and Industrial Engineering, College of Engineering, Majmaah University, Al-Majmaah 11952, Saudi Arabia; saldahash@mu.edu.sa; 2 Department of Mechanical Design and Production Engineering, Faculty of Engineering, Assiut University, Assiut 71515, Egypt

**Keywords:** selective laser sintering, polyamide 12, carbon fibers, graphite, friction and wear, dry sliding

## Abstract

Carbon fiber-reinforced polymers (CFRPs) are being used extensively in modern industries that require a high strength-to-weight ratio, such as aerospace, automotive, motorsport, and sports equipment. However, although reinforcement with carbon fibers improves the mechanical properties of polymers, this comes at the expense of abrasive wear resistance. Therefore, to efficiently utilize CFRPs in dry sliding contacts, solid lubricant is used as a filler. Further, to facilitate the fabrication of objects with complex geometries, selective laser sintering (SLS) can be employed. Accordingly, in the present work, graphite-filled carbon fiber-reinforced polyamide 12 (CFR-PA12) specimens were prepared using the SLS process to explore the dry sliding friction and wear characteristics of the composite. The test specimens were aligned along four different orientations in the build chamber of the SLS machine to determine the orientation-dependent tribological properties. The experiments were conducted using a pin-on-disc tribometer to measure the coefficient of friction (COF), interface temperature, friction-induced noise, and specific wear rate. In addition, scanning electron microscopy (SEM) of tribo-surfaces was conducted to specify the dominant wear pattern. The results indicated that the steady-state COF, contact temperature, and wear pattern of graphite-filled CFR-PA12 are orientation-independent and that the contact temperature is likely to approach an asymptote far below the glass transition temperature of amorphous PA12 zones, thus eliminating the possibility of matrix softening. Additionally, the results showed that the Z-oriented specimen exhibits the lowest level of friction-induced noise along with the highest wear resistance. Moreover, SEM of tribo-surfaces determined that abrasive wear is the dominant wear pattern.

## 1. Introduction

Selective laser sintering (SLS), one of the most popular 3D printing techniques, is a layer-by-layer manufacturing process in which laser energy is used to fuse the particles of a thermoplastic powder in selected areas that match the layer cross-section of the object 3D model. The unfused powder provides support for the object being printed, thus eliminating the need for additional supporting structure, which makes SLS a favorable technique for additive manufacturing of objects with complex geometries [1]. In addition to being used for a long time as a rapid prototyping technique, SLS is currently being used to produce small-to-medium-sized patches of polymeric objects. 

Among the few pure materials suitable for the SLS process, polyamide 12 (PA12) is considered the most popular thermoplastic powder because of its favorable sintering properties, which include good powder flowability, low melt viscosity, and a wide range between melting and crystallization temperatures [2]. Even though PA12 parts prepared by the SLS process have high chemical/environmental stability, reasonable mechanical properties, high impact resistance, and acceptable wear resistance, the application of SLS to modern industries calls for composite materials based on PA12 with tailored mechanical and tribological properties [3,4,5]. Therefore, fillers in the form of either particulates, fibers, or a combination of both have been introduced to enhance the mechanical and/or tribological properties of laser-sintered functional parts. The most common particulate fillers include graphite, molybdenum disulfide (MoS_2_), polytetrafluoroethylene (PTFE), boron nitride (BN), and glass beads [6,7], while carbon fibers, glass fibers, and aramid fibers are the most widely used synthetic fibers for the reinforcement of polymer matrix [8]. 

Recently, polymeric composites have proven to be good replacements for traditional materials in modern applications that require a high strength-to-weight ratio, such as aerospace, automotive, motorsport, and sports equipment. Hence, due care must be paid when designing the polymer composite for a specific application. Proper selection of particulate/fiber material, shape and size, weight ratio, and surface treatment is extremely important. The properties of fiber-reinforced polymer composites are extremely sensitive to fiber weight fraction, fiber length, fiber orientation, fiber preprocessing, and fiber/matrix adhesion [9,10]. As the properties of SLS-fabricated parts are orientation-dependent [11,12,13], it was found that reinforcement with either particles or short fiber enhances the homogeneity of polymer composites prepared by the SLS process [14,15], while introducing long or continuous fibers as a reinforcement results in directional properties of the fibrous composite [9]. Further, the ease with which short fibers are processed saves manufacturing costs considerably, making them most suitable for the reinforcement of polymer matrices in additive manufacturing processes [16]. Among the short fibers used for reinforcement purposes, carbon fibers (CF) are the most widely used fibers to improve the mechanical properties of polymer composites used in the automotive and motorsport industries. Furthermore, as neat polymers are thermal insulators, reinforcement with carbon fibers enhances the thermal conductivity of polymeric composites significantly. Nevertheless, although reinforcement of the thermoplastic matrix with short carbon fibers improves the tensile strength and wear resistance, decreases the coefficient of friction and frictional heating, and enhances the thermal conductivity of thermoplastic composites, it was found that the elongation at break has decreased significantly [17,18,19]. Accordingly, the abrasive wear resistance of carbon fiber-reinforced polymers (CFRPs) is unsatisfactory. Moreover, in dry sliding conditions, it was reported that fiber crushing results in fiber thinning followed by fiber pull-out of the matrix, which in turn results in unstable frictional properties [20,21]. Therefore, reinforcement with short carbon fibers can efficiently promote the mechanical rather than the tribological properties of thermoplastic polymers. As short carbon fiber-reinforced polymers are being used in applications where they are prone to direct contact with metallic/polymeric counterparts, it becomes inevitable to improve the friction and wear characteristics of such composites. For this purpose, solid lubricants in the form of particulate fillers have been employed to boost the lubricity of carbon fiber-reinforced polymers. 

Graphite is a popular solid lubricant that has been extensively used to improve the lubricity of tribo-surfaces. The lubricating effect of graphite stems from its distinctive layered crystal structure, in which carbon atoms are bonded covalently in a hexagonal pattern within each layer, and layers are bonded to each other by means of weak Van der Waals forces. As a result, the bulk shear strength of graphite is always lower than the interfacial shear resistance, allowing graphene layers to be easily removed and transferred to the counter-surface in sliding contact, thereby reducing the coefficient of friction and improving the wear resistance of the matrix [22,23]. In addition, graphite as a filler enhances the mechanical properties and thermal conductivity of the polymer matrix [22]. Hence, to improve the tribological properties of the thermoplastic matrix and maintain prominent mechanical properties, graphite powder can be introduced as a filler for carbon fiber-reinforced polymers. By combining the effects of graphite as a solid lubricant and short carbon fibers as a reinforcement of the thermoplastic matrix, the resulting composite can have enhanced tribological as well as mechanical properties. 

With PA12 as a thermoplastic matrix, graphite-filled carbon fiber-reinforced PA12 composite can meet the increasing demand for a stiff, lightweight, thermally conductive, low-friction, wear-resistant, and chemically stable material for aircraft, automotive, and Motorsport industries. The composite can be ideal for applications with fixed and movable joints operating at low temperatures (less than the glass transition temperature of PA12) and under a wide range of bearing pressures in harsh environments. Moreover, with graphite powder, short carbon fibers, and PA12 as the constituents, the composite is most suitable for SLS processing, which facilitates the fabrication of complex functional geometries based on this composite. However, so far, very little has been reported regarding the tribological characteristics of a reinforced thermoplastic filled with a solid lubricant, and very few articles among them are concerned with composites prepared by the SLS technique [15,24,25,26]. The few reported results showed that inclusion of either PTFE or MoS_2_ could effectively reduce the coefficient of friction of laser-sintered PA12 by forming a homogenous transfer film on the counter-surface [15,27], and that a 5–10% weight fraction of graphite was sufficient to significantly improve the tribological characteristics of PA6 [24].

Hence, this experimental study is dedicated to exploring the tribological features of graphite-filled carbon fiber-reinforced PA12 (CFR-PA12) composites prepared by the SLS process. Given that the properties of SLS parts are orientation-dependent, the test specimens are aligned along four orientations in the build chamber of the SLS machine. The tribological characteristics of graphite-filled CFR-PA12 composites are examined using a pin-on-disc tribometer in dry sliding conditions. Frictional heating as well as the friction-induced noise are measured, and the wear resistance of the composite is evaluated. Finally, scanning electron microscopy (SEM) is used to investigate the wear patterns that dominate the worn tribo-surface of test specimens. The results from this study, when compiled with relevant findings in the open literature, can contribute to a better understanding of the tribological characteristics of graphite-filled CFR-PA12 composite prepared by SLS under dry sliding conditions; this can facilitate widening the applicability of this composite in demanding industries.

## 2. Materials and Methods

### 2.1. Selective Laser Sintering of Test Specimens

Selective laser sintering was used to fabricate the test specimens because the SLS process has proven to be the most reliable 3D printing technology suitable for the manufacturing of objects with complex geometries from a wide range of thermoplastic powders. Further, during the SLS process, the unsintered powder supports the part being manufactured, thus eliminating the need for supporting structures. Moreover, the SLS system is advantageous because it is able to manufacture parts with tailored properties through the proper selection of fabrication parameters (laser power, scanning speed, scan spacing, and layer thickness). Another prominent feature of the SLS system is its relatively low manufacturing cost, as the unsintered powder can be reused in subsequent fabrication processes. Hence, the test specimens were prepared by the SLS technique using a powder mixture composed of polyamide 12 (PA12) powder as a matrix, a 5% weight ratio of graphite powder, and a 2.0% weight ratio of short carbon fibers. The powder mixture was developed in-house by Graphite Additive Manufacturing Ltd. (Aylesbury, UK) and supplied by EOS GmbH (Düsseldorf, Germany). Hence, micro-sized graphite powder was used as a particulate filler, while short carbon fibers with 8–10 μm diameter and 20–60 μm in length were used as reinforcement of PA12 matrix; it was reported that carbon fiber length of 10–100 μm is optimum for the SLS process [28].

**Figure 1:**
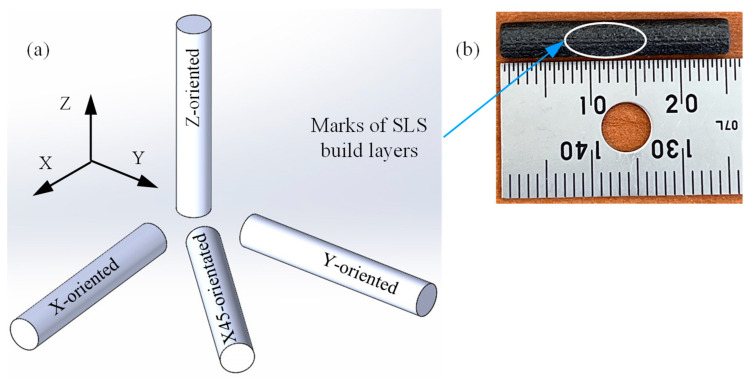
(**a**) Schematics of specimens’ orientation in the SLS build chamber. (**b**) X-oriented test specimen showing marks of build layers.

Specimens in the form of pins with a diameter of 4 mm and a length of 25 mm were fabricated in the SLS build chamber in four different orientations. Fabrication parameters are given in Table 1. The used SLS system (3D Systems sPro 60 HD-HS) is equipped with a CO_2_ laser source of 70 W and a wavelength of 10,600 nm. The specimens were oriented along the X-axis, Y-axis, Z-axis, and at 45° to the X-axis (X45) in the build chamber, as shown in Figure 1a, and were built along the positive Z-axis. The tribo-surfaces of test specimens are perpendicular to each orientation. It is worth noting that the SLS fabrication parameters outlined in Table 1 were selected by the manufacturer for optimum part density, dimensional accuracy, and mechanical properties. Cosmetic finishing (light media blasting) was applied to remove the unsintered powder particles from the specimen surface after drawing them out of the build chamber. Figure 1b shows clear marks of SLS build layers in the X-oriented specimen; however, this was not visible on the surface of other specimens. 

### 2.2. Pin-on-Disc Tribometer

**Figure 2:**
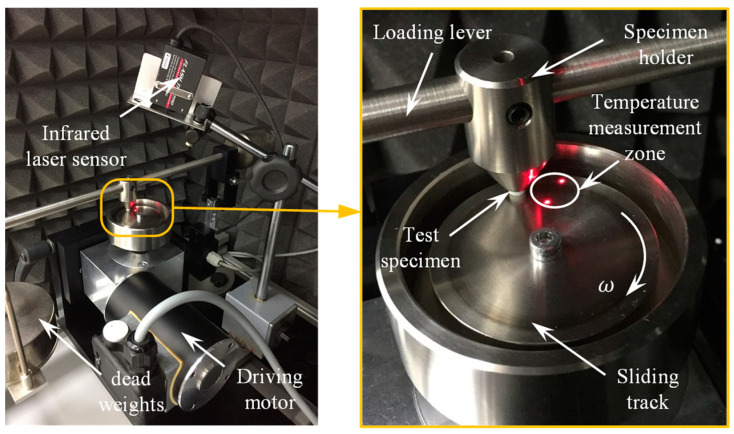
Experimental test rig using a pin-on-disc tribometer [7,20].

The dry sliding characteristics of graphite-filled CFR-PA12 composite were investigated using a pin-on-disc tribometer, at which the pin-shape test specimen is inserted into a pin holder located in the middle of a loading lever. Then, the pin is loaded against the steel disc by means of a hanging deadweight at the end of the loading lever (see Figure 2). The stainless steel disc is rotated by means of a drive unit while the specimen is fixed. To maintain dry sliding conditions at the interface, 99.9% ethanol was used to clean the disc surface to remove any dirt, oily substances, or adsorbed matter, and the experiments were conducted in a dry environment (air humidity was less than 10%). Several stainless steel discs were manufactured with close surface roughness so that every test specimen is examined against fresh disc tribo-surface; this was adopted to rule out the effect of the formed transfer film on friction and wear behavior at the interface. The surface roughness of steel discs was measured using portable surface roughness tester (SURFTEST SJ-210, Mitutoyo, Kanagawa, Japan), and the average surface roughness (Ra) value in the radial direction (i.e., perpendicular to the sliding direction) of all disc surfaces was 0.34 ± 0.03 microns.

### 2.3. Experimental Measurements

The measurements were conducted to evaluate the coefficient of friction (COF), approximate contact temperature, friction-induced noise, specific wear rate, and to investigate the dominant wear pattern along each build orientation. To evaluate the COF at the composite/disc interface, the friction force was measured by means of a double-bending beam force sensor along with an NI-9237 module and an NI-compactDAQ controller [7,20]. A Labview code was developed to manage the sensor signal, in which a moving average function was used to return the average value of the COF every 0.5 s. 

Since measurement of the actual contact temperature is not straightforward, a close approximation of contact temperature was obtained by measuring the disc temperature very close to the trailing edge of the pin (see Figure 2). For this purpose, an infrared laser temperature sensor (FT-H30, KEYENCE, Itasca, IL, USA) and a digital amplifier unit (FT-50AW, KEYENCE, Itasca, IL, USA) were used to detect the frictional heat emitted from a circular area of 6 mm in diameter in the vicinity of the contact zone. To measure the friction-induced noise, the pin-on-disc tribometer was arranged inside a semi-anechoic chamber lined with high-density polyurethane acoustic foam. The noise level was measured using a digital sound level meter (GM 1357, Benetech co., Shenzhen, China). An Arduino Uno microcontroller board (SMD R3, Arduino.cc, Ivrea, Italy) was used to manage the output signals of the infrared laser temperature sensor and the digital sound level meter, and a moving average function was applied to calculate the average values of contact temperature and frictional noise level every 0.5 s. 

Finally, a thin film of gold (less than 100 Å) was deposited on the worn tribo-surfaces of graphite-filled CFR-PA12 specimens in a plasma sputtering device (sec-MCM-100P ion sputtering coater) to render them electrically conductive, and then scanned with a scanning electron microscope (SNE-4500M Plus, Sec Co., Suwon, Republic of Korea) to determine the wear patterns that predominated the tribo-surface.

### 2.4. Experimental Conditions

The results presented in this work outline the tribological characteristics of a graphite-filled CFR-PA12 composite prepared using SLS under dry sliding conditions against a steel counter surface. Tribological characterization was conducted using a pin-on-disc tribometer, where the pin is a rod with a diameter of 4 mm and length of 25 mm and made of laser-sintered graphite-filled CFR-PA12 composite, while the disc is made of stainless steel. The applied load on the pin was 50 N, thus the apparent contact pressure at the pin/disc interface was about 4 MPa; however, the actual contact pressure is much higher than the apparent value as the real contact area is much smaller than the apparent one [29]. The sliding track radius was 20 mm, and the disc rotation velocity was 120 rpm; thus, the pin sliding speed was about 250 mm/s. A dry sliding experiment was conducted for a duration of 45 min to explore the steady-state performance of the graphite-filled CFR-PA12 composite. The current experimental conditions are given in Table 2.

## 3. Results and Discussions

The results presented in the following sections address the potential effects of part orientation on the coefficient of friction, friction-induced noise, contact temperature, and specific wear rate of laser-sintered graphite-filled CFR-PA12 composites under dry sliding conditions against steel countersurfaces.

### 3.1. Coefficient of Friction (COF)

**Figure 3:**
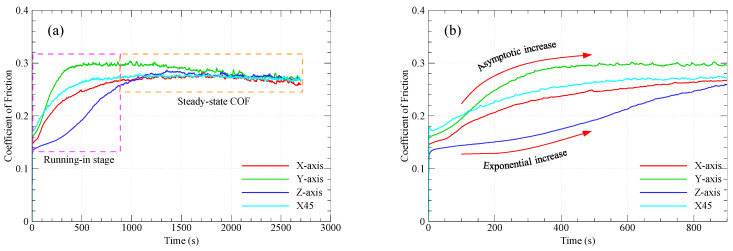
(**a**) Variations of the COF with sliding time. (**b**) COF increase patterns during the running-in stage.

**Figure 4:**
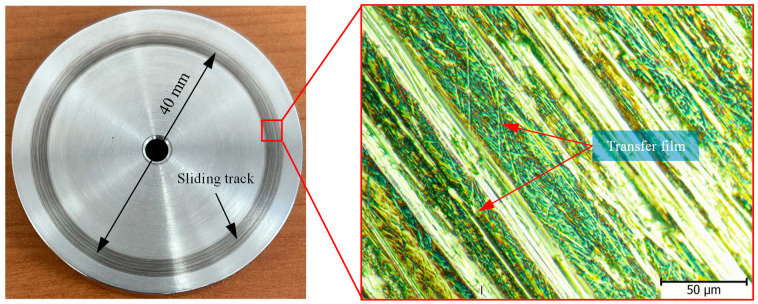
Formation of transfer film on the disc surface (optical microscopy).

There are two components of friction: the mechanical component representing the material resistance to ploughing/microcutting by the asperities of the counter surface, and the adhesion component representing the resistance to shearing of adhesive junctions at spots of real contact. Indeed, the adhesion component contributes most to the frictional resistance of polymers and polymer composites [30,31]. Figure 3 shows the variations of the COF over sliding time for graphite-filled CFR-PA12 specimens with different orientations. The results from Figure 3a reveal that there has been a sharp increase in the COF of X-oriented, Y-oriented, and X45-oriented specimens during the running-in stage, while the Z-oriented specimen shows a gradual increase in COF during the same process. The running-in process lasts for a few minutes (less than 10 min), then a steady state is attained at which the COF of all specimens is remarkably close. Interestingly, the steady-state COF of all specimens is about 0.26 and is likely to fall behind this value as sliding continues. Such low COF indicates that abrasive wear dominates the tribo-surface of graphite-filled CFR-PA12 specimens, regardless of part build orientation. Indeed, reinforcing PA12 with short carbon fibers decreases the elongation at break considerably and, consequently, reduces the abrasive wear resistance of CFR-PA12. Additionally, introducing graphite powder as a filler further decreases the elongation at the beak and hence the abrasive wear resistance of the specimens, according to the Ratner-Lancaster correlation [31]. Due to the lamellar structure of graphite, upon sliding against a steel surface, the weak Van der Waals forces of attraction between layers allow graphite layers to be easily sheared away and transferred into the disc surface, thus increasing the interface lubricity and decreasing the steady-state COF. Figure 4 shows evidence of a transfer film attached to the sliding track on the disc surface after testing graphite-filled CFR-PA12 samples. Consequently, transferred graphite layers on the disc surface decrease the interfacial shear strength of adhesive junctions, rendering all junctions to be sheared away at the interface rather than at the polymer subsurface, and hence the frictional heating, friction-induced noise, and wear volume are decreased significantly.

The gradual increase of the COF for Z-oriented specimens at the running-in stage, shown in Figure 3b, is attributed to the corresponding gradual increase in real contact area. At the initial stages of the running-in process, carbon fibers contribute to supporting the normal load; however, as rubbing continues, the PA12 layer that encapsulates the fibers and graphite particles is continuously removed, rendering the fibers and graphite particles susceptible to direct contact with the disc surface. Consequently, thinning of carbon fibers occurs and more PA12 spots get into contact with the disc surface, resulting in a corresponding increase in adhesive resistance [20], hence the COF of the Z-oriented specimen increases accordingly.

Contrarily, the steep increase in COF of specimens built along X, Y, and X45 directions during the running-in process is attributed to the frequent formation and shearing out of adhesive junctions at the sliding interface since it was reported that most frictional resistance is associated with adhesion at the interface until a stable transfer film of graphite layers is formed on the disc surface, then a steady state COF is reached and manifested by a uniform wear rate. Indeed, the highest wear volume of polymer composites is associated with the running-in process [32]. 

### 3.2. Friction-Induced Noise

**Figure 5:**
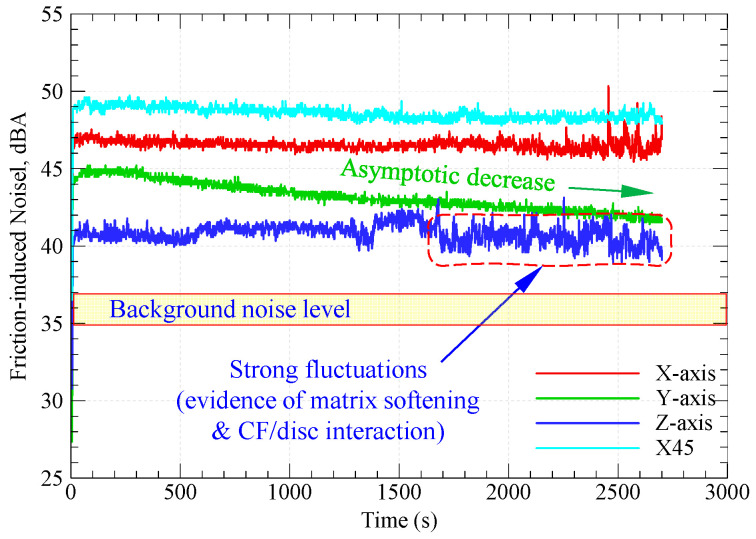
Variations of friction-induced noise with sliding time for specimens with different orientations.

In polymer tribology, friction-induced noise is attributed to the adhesive component of friction at the sliding interface. Adhesion bonds are formed either at surface or subsurface zones of real contact. Surface adhesive bonds are formed when the gap size between polymer and countersurface is less than 100 Å by means of weak Van der Waals molecular forces. Meanwhile, subsurface adhesive bonds are formed at both sides of ploughing/cutting asperities of the harder surface. In both cases, the breaking of adhesive bonds results in what is termed friction-induced noise, which is a ruling parameter in polymer tribology. Therefore, by reducing the COF, the friction-induced noise is reduced significantly. The most effective way to reduce the adhesive component of friction is to lubricate the interface, either using liquid or solid lubricant. Figure 5 shows frictional noise variations with sliding time for graphite-filled CFR-PA12 specimens of various orientations. It is worth noting that the background noise at the experimentation site was 35–37 dBA. 

**Figure 6:**
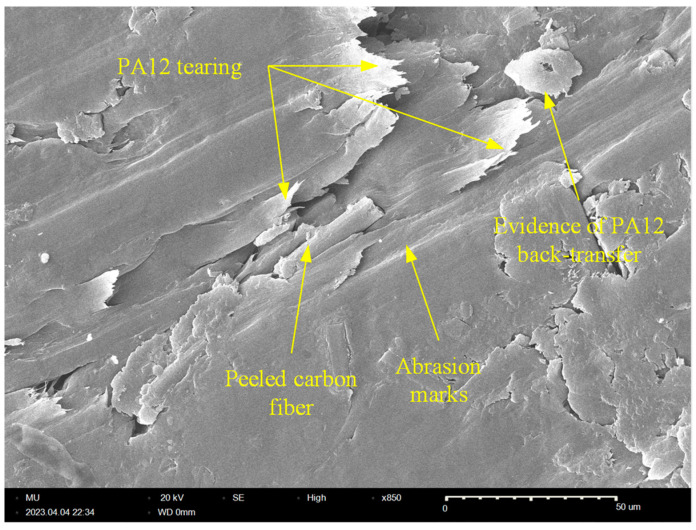
SEM (850×) of the worn surface Z-oriented specimen shows marks of adhesive wear.

Although the steady-state COF of all specimens is remarkably close, the results from Figure 5 depict that the test specimens have diverse levels of friction-induced noise throughout the test duration. A possible explanation for this inconsistency might be that the pattern of friction at the running-in stage has a prolonged effect on the friction-induced noise. The repeated formation/breaking of adhesive junctions between X-, Y-, and X45-oriented specimens and disc surfaces results in steep growth of both the COF and friction-induced noise at the running-in stage. It is apparent from Figure 3b and Figure 5 that, during the running-in stage, the COF increases in an asymptotic manner and so does the friction-induced noise until steady state conditions are attained, and then the friction noise remains at the same level throughout the experiment duration. Another interesting finding from Figure 4 and Figure 5 is the remarkable synchronous pattern of evolution of friction-induced noise and COF for the Y-oriented specimen, at which the friction-induced noise asymptotically decreases as the steady-state COF decreases. Another important remark from Figure 5 is that the Z-oriented specimen has the lowest friction noise level, but with severe fluctuations. Since the COF of the Z-oriented specimen is still near the lowest value amongst all orientations throughout the test duration, this behavior may be attributed to the interaction between carbon fibers and disc surface [20]; however, this explanation does not rule out the prominent effect of interfacial adhesion as the real area of contact increases with increasing sliding time. To emphasize the role of interfacial adhesion, Figure 6 shows clear marks of strong tearing of the PA12 matrix, manifested by the appearance of stringy tongues unfirmly attached to the surface. For tearing to occur, strong interfacial adhesion along with anisotropic cohesion properties must exist. Tearing results in the formation of PA12 wear flakes that may roll between surfaces or be attached to a sliding surface [33,34,35]. Similarly, the friction-induced noise of an X-oriented specimen tends to exhibit strong fluctuations near the end of testing time. This may be influenced by the softening of the PA12 matrix because of the cumulative frictional heating that fosters the formation of adhesive bonds, which results in an increase in frictional noise (see Figure 6).

### 3.3. Contact Temperature

**Figure 7:**
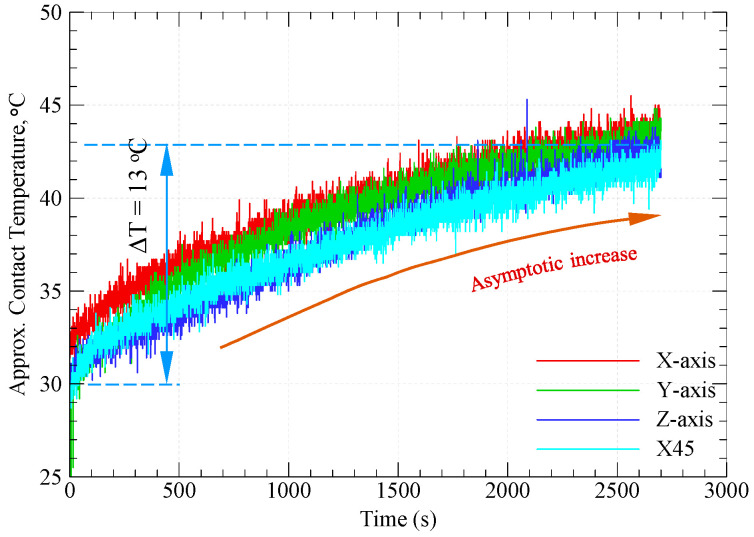
Asymptotic increase of contact temperature with sliding time.

When a thermoplastic slides against a hard counter surface, the interfacial interaction results in frictional energy losses in one or more of the following forms: (1) losses due to subsurface plastic deformations by means of hard asperities (ploughing and/or microcutting); (2) losses due to surface adhesion; (3) losses due to subsurface adhesion in ploughing and/or microcutting; and (4) losses due to polymer hysteresis (difference between induced elastic deformation energy and the retained back-pushing elastic energy). Indeed, ploughing and microcutting are principal sources of subsurface heating, while adhesion can be a major source of surface as well as subsurface heating. Meanwhile, hysteresis losses contribute to subsurface heating. Moreover, repeated formation/rupture of adhesive bonds causes severe vibrations of subsurface polymer molecules that contribute to subsurface heating [36]. Figure 7 shows the variations in approximate contact temperature with sliding time for specimens built along the four different orientations. It is apparent from Figure 7 that the approximate contact temperature increases in an asymptotic manner with increasing sliding time; however, there is no significant difference between the contact temperatures of different specimens. Further, the cumulative increase in contact temperature is only 13 °C after 45 min of dry sliding. Indeed, the enhanced thermal conductivity of graphite and carbon fibers contributes to the thermal stability of graphite-filled CFR-PA12 composites, regardless of build orientation. Additionally, the transfer film of graphite layers that formed on the disc surface acts to decrease surface and subsurface adhesion, which is the major source of frictional heating in dry sliding conditions. The most interesting result from Figure 7 is that increasing the sliding time is unlikely to cause a corresponding increase in contact temperature, as the contact temperatures tend to reach an asymptote far below the glass transition temperature (Tg) of the amorphous regions of the PA12 matrix. In fact, introducing CFs as a reinforcement is expected to increase the glass transition temperature of PA12. This can be explained as follows: upon sliding, the CFs support the majority of the applied load, and hence a high contact pressure is developed at the disc/CFs interface, which in turn is transmitted to the PA12 matrix. As the crystallinity percent of laser-sintered PA12 is 24.5% [37], high levels of contact pressure result in a corresponding increase in glass transition temperature in amorphous regions of the PA12 matrix. Accordingly, since the glass transition temperature of PA12 is around 50 °C [38,39], including graphite and carbon fibers is likely to increase the glass transition temperature beyond this value. As the contact temperature of graphite-filled CFR-PA12 increases in an asymptotic manner that is far below 50 °C, the result from Figure 7 demonstrates the potential of this composite to replace conventional bearings operating in harsh environmental conditions.

### 3.4. Specific Wear Rate

Evaluation of a material’s specific wear rate is an efficient method to visualize its wear resistance; wear resistance is proportional to the reciprocal of the specific wear rate. The specific wear rate of polymer composites (*Ks*) depends on the wear volume (Δ*V*), normal load (*F_N_*), and sliding distance (*L*) and is expressed as [40]:

(1) $$K_{s}=\frac{∆V}{F_{N}\times L}$$

**Figure 8:**
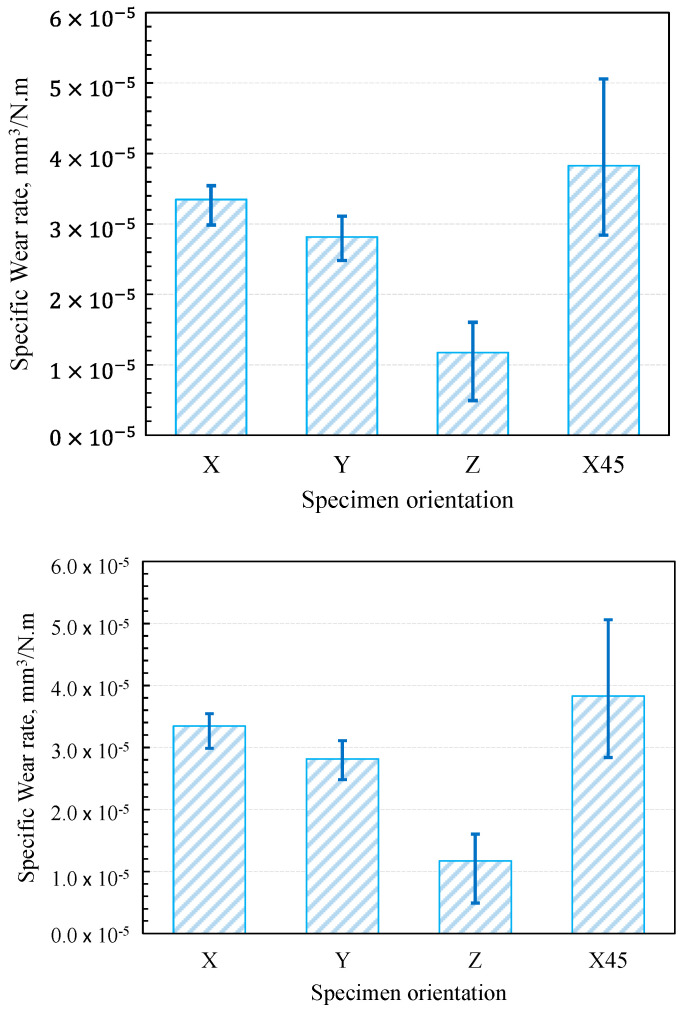
Specific wear rate of graphite-filled CFR-PA12 specimens with different orientations.

Figure 8 shows the specific wear rates of graphite-filled CFR-PA12 specimens built along the four different orientations. The results reveal that the Z-oriented specimen has the lowest specific wear rate and hence the highest wear resistance amongst all other orientations. Accordingly, the results from Figure 8 are in good agreement with those obtained from Figure 3, Figure 4, Figure 5, Figure 6 and Figure 7, as the Z-oriented specimen exhibited the lowest levels of COF and friction-induced noise. However, it is somewhat surprising that the X45-oriented specimen shows a comparatively high specific wear rate and hence a lower wear resistance, although it has a comparable COF to other specimens. A possible explanation for this behavior might be the continuous removal of the transfer film from the disc surface; it was found that the wear rate of polymers is dictated by the rate of removal of the transfer film from the countersurface rather than by the rate of polymer transfer into the film [31]. The transfer film can be removed from the disc surface by the peeling action of protuberant carbon fibers [20]; this is manifested by the detected high level of frictional noise of the X45-oriented specimen (see Figure 5). Likewise, the high frictional noise can be evidence of strong surface and/or subsurface adhesion that leads to localized tearing of the PA12 matrix and subsequent buildup of the transferred layer on the disc surface. Eventually, the repeated rubbing causes such lumps of wear debris to be detached and replaced by a fresh migrating film. Such poorly attached transferred film is the main reason behind the increased wear rate of the X45-oriented specimen.

Nevertheless, it is important to keep in mind that the tribological characteristics of thermoplastic composites are susceptible to small variations in experimental conditions. Therefore, the abovementioned results cannot be extrapolated to conditions other than those mentioned in Table 2. Hence, thorough investigations for a wide range of experimental conditions combined with a proper machine learning (ML) model can help in fabricating components with customized tribological properties based on graphite-filled CFR-PA12 composite [5]. Additionally, the results from Figure 3, Figure 4, Figure 5, Figure 6, Figure 7 and Figure 8 suggest that functional sliding bearings based on graphite-filled CFR-PA12 composite where the bearing surface is built normal to the Z-orientation can perform reliably under low sliding speeds and high contact pressure; such prominent features render the laser-sintered graphite-filled CFR-PA12 composite ideal for aerospace, motorsport, and electric vehicle applications.

### 3.5. Scanning Electron Microscopy (SEM)

**Figure 9:**
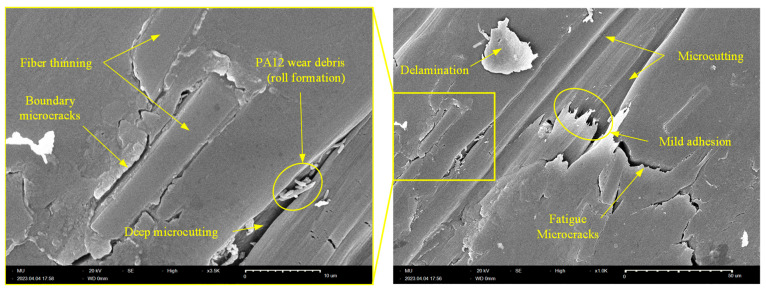
SEM of the worn tribo-surface of the X-oriented graphite-filled CFR-PA12 specimen.

The worn tribo-surfaces of the specimens were investigated using SEM to determine the prevailing wear patterns for the purpose of suggesting microstructural solutions to enhance the wear resistance of the composite. Figure 9 shows an SEM picture of the worn surface of the X-oriented specimen. The tribo-surface shows evidence of weak adhesion and is characterized by clear marks of abrasive wear in the form of deep microcutting grooves. Further, the tribo-surface of the X-oriented specimen features two types of microcracks: (1) boundary microcracks at the CF/PA12 interface due to poor bonding that necessitates proper surface treatment of CF to enhance adhesion with the PA12 matrix; and (2) fatigue microcracks (aligned normal to the sliding direction) due to repeated shearing of adhesive junctions at the interface. Additionally, fiber thinning was detected, which supports the hypothesis that disc/fiber interaction contributes to the friction-induced noise of carbon fiber-reinforced polymers (CFRPs). Moreover, the existence of tiny rolls of PA12 wear debris (i.e., roll formation) that are collected in deep microcuts is evidence of weak interfacial adhesion. Furthermore, the tribo-surface of the X-oriented specimen shows a loosely attached PA12 lump as evidence of delamination. The delamination of the PA12 matrix occurs as a result of the propagation and subsequent coalescence of surface microcracks (either fatigue microcracks or boundary cracks).

**Figure 10:**
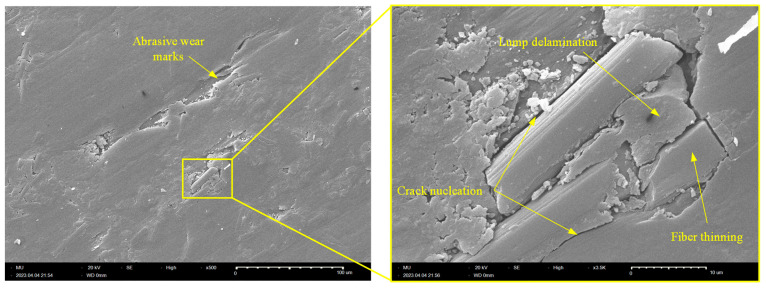
SEM of the worn tribo-surface of the Y-oriented graphite-filled CFR-PA12 specimen.

Similarly, Figure 10 shows an SEM picture of the worn surface of the Y-oriented specimen. The results from Figure 10 indicate that abrasive wear is the dominant wear pattern of the Y-oriented tribo-surface, which is free from any signs of interfacial adhesion. Another interesting feature of the Y-oriented CF-PA12 tribo-surface is fiber thinning by the pulverization action of the steel countersurface, which releases fine graphite particles and further lubricates the composite/disc interface. Eventually, released graphite particles from both sources (i.e., shearing of graphite inclusions and pulverization of CFs) act to decrease the COF, friction-induced noise, and interface temperature. This may explain the remarkable asymptotic decrease of both the COF and the friction-induced noise of the Y-oriented specimen. Further analysis of Figure 10 reveals that upon coalescence of boundary cracks at the CF/PA12 interface, delamination occurs and releases lumps of PA12 that may initiate a state of three-body abrasion [41,42].

**Figure 11:**
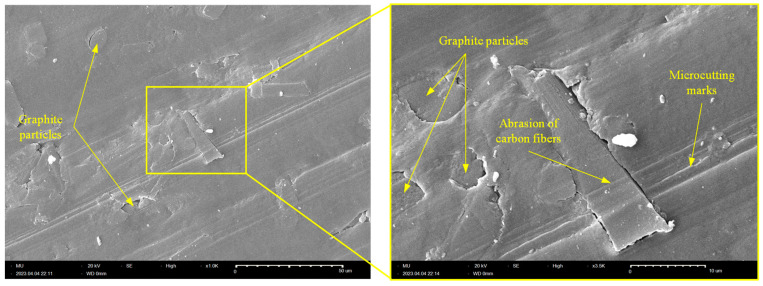
SEM of the worn tribo-surface of the Z-oriented graphite-filled CFR-PA12 specimen.

Likewise, Figure 11 further confirms that abrasive wear is the dominant wear pattern of the Z-oriented graphite-filled CFR-PA12 specimen; the tribo-surface is characterized by a continuous microcutting groove. In addition, the tribo-surface shows straightforward evidence of CF pulverization and shearing/chopping of graphite filler. Although thinning/pulverization of CFs produces further graphite powder that effectively reduces the COF and wear rate, this comes at the expense of frictional noise as the prolonged interaction between CFs and disc surface eventually increases the friction-induced noise of Z-oriented specimens (see Figure 5).

**Figure 12:**
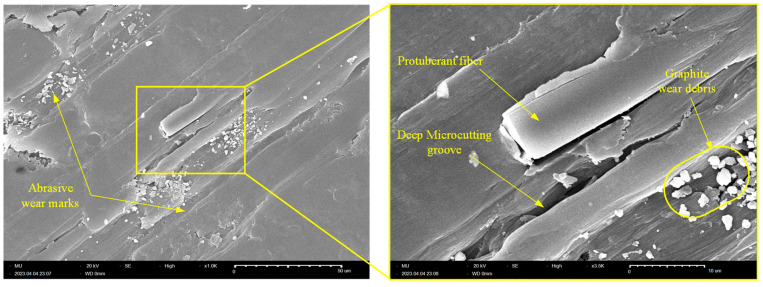
SEM of the worn tribo-surface of the X45-oriented graphite-filled CFR-PA12 specimen.

Similarly, Figure 12 shows an SEM picture of the tribo-surface of an X45-oriented specimen, where the worn surface features clear marks of deep microcutting grooves. Additionally, tiny graphite debris, which formed as a result of either CF crushing or shearing of graphite filler particles, is noticeable. However, the most interesting features of the tribo-surface of the X45-oriented specimen are the presence of protruded CFs and the deep microcutting grooves; these two features were the reason behind the friction and wear characteristics of the X45 specimen, as the protuberant carbon fibers that are aligned parallel to the sliding direction act to remove the transfer film from the disc surface, hence increasing the specific wear rate of the X45-oriented specimen. Further, the interaction of CF with the disc surface increases the friction-induced noise as CFs are stiff amorphous materials [20]. Meanwhile, the deep microcutting groove affirms the possibility of subsurface adhesion at either side of the cutting asperities and hence contributes to the elevated level of frictional noise.

## 4. Conclusions

The present experimental study was conducted to explore the dry friction and wear characteristics of graphite-filled CFR-PA12 composites that were prepared by the SLS process. The effect of part-build orientation on tribological properties was investigated. For this purpose, the COF, contact temperature, friction-induced noise, and wear rate were measured for specimens built along four different orientations (X, Y, Z, and X45 orientations). The experiments were conducted using a pin-on-disc tribometer, and scanning electron microscopy (SEM) was used to investigate the wear patterns that dominated the tribo-surfaces. The results revealed that the steady-state COF, contact temperature, and wear pattern of graphite-filled CFR-PA12 are orientation-independent and that abrasive wear is the dominant wear pattern regardless of build orientation. In addition, the results showed that the Z-oriented specimen possesses the highest wear resistance combined with the lowest level of frictional noise among all other orientations. Unlike other orientations, the COF of Z-oriented specimens increases in an exponential manner during the running-in stage as a result of CFs/disc interactions. As a result of CFs/disc interaction, fiber crushing and/or thinning occurs, which eventually releases fine graphite particles between the rubbing surfaces and thus enhances the interface lubricity.

Furthermore, analysis of tribo-surfaces using SEM determined that shearing of graphite inclusions and fibers thinning release graphite debris at the interface, thus decreasing the COF, contact temperature, and friction-induced noise. Optical microscopy of the disc surface showed that a stable transfer film was formed on the disc surface that acts to decrease the COF, surface and subsurface adhesion, and specific wear rate of the Z-oriented test specimen.

However, despite the abovementioned promising features of the laser-sintered graphite-filled CFR-PA12 composite, further research is needed to explore its tribological properties under a wide range of operating conditions. With this in hand, artificial intelligence can be employed to help fabricate functional bearings with customized tribological properties.

## Figures and Tables

**Table 1:** SLS parameters of graphite-filled CF-PA12 composite.

	Graphite/CF/PA12 Composite
Powder color	Black color
SLS system	3D Systems SLS sPro 60 HD-HS
Outline power (W)	11
Hatching power (W)	34
Scanning speed (m/s)	12
Scan spacing (mm)	0.15
Layer thickness (mm)	0.1

**Table 2:** Pin-on-disc experimental conditions of graphite-filled CFR-PA12 composite.

	Specifications
Normal load (N)	50
Disc rotation velocity (rpm)	120
Sliding track radius (mm)	20
Sliding speed (mm/s)	250
Test duration (min)	45
Disc initial temperature (°C)	29–30
Background noise level (dBA)	35–37
Humidity (%)	7–10

## Data Availability

The data are contained within the article.
